# Optimized Protocol for Isolation and Culture of Murine Neonatal Primary Lung Fibroblasts

**DOI:** 10.3390/mps6010014

**Published:** 2023-01-24

**Authors:** Rocío Fuentes-Mateos, Eugenio Santos, Alberto Fernández-Medarde

**Affiliations:** Centro de Investigación del Cáncer, Instituto de Biología Molecular y Celular del Cáncer, Universidad de Salamanca/CSIC and CIBERONC, 37007 Salamanca, Spain

**Keywords:** primary lung fibroblasts isolation, neonatal mice, cell culture, lung mesenchyme isolation, murine primary lung fibroblast culture

## Abstract

During all the stages of lung development, the lung mesoderm (or mesenchyme) is closely related to the endoderm, and their cross-regulation promotes, controls, and drives all lung developmental processes. Generation of 3D organoids in vitro, RNA assays, and mitochondrial respiration studies are used to analyze lung development and regeneration to better understand the interactions between epithelium and mesenchyme, as well as for the study of redox alterations and the metabolic status of the cells. Moreover, to avoid using immortalized cell lines in these in vitro approaches, standardized murine neonatal primary lung fibroblast isolation techniques are essential. Here, we present an optimized method to isolate, culture, and freeze primary lung fibroblasts from neonatal lungs. Our current method includes step-by-step instructions accompanied by graphical explanations and critical steps.

## 1. Introduction

Lung mesoderm proliferation and differentiation must be coordinated with the endoderm through complex signaling networks to give rise to a different subset of differentiated epithelial cells that form the mature lung [1,2,3]. Alterations in this critical interaction can lead to severe anatomical and functional defects in the airway and alveoli, linked to high neonatal mortality in humans [4,5,6]. Hence, analyses of lung mesenchyme biology and function are indispensable to provide knowledge about the role of endogenous lung fibroblasts in several physiological and pathological conditions, as they are implicated in the regenerative potential of the lung epithelium [7,8,9,10]. Understanding the lung mesenchyme impact during lung development, as well as in lung diseases such as idiopathic pulmonary fibrosis (IPF) [11], emphysema [12], or chronic obstructive pulmonary disease (COPD) [13,14] will allow the discovery of new molecular targets used for the development of new therapeutic tools for those diseases.

In this regard, mouse models mimicking these conditions and cell cultures derived from them are useful tools for studies on human illnesses [15]. However, we lack efficient protocols for the isolation and culture of primary fibroblast from newborn lungs, and the existing protocols developed for adult lungs are not suitable [16,17]. In addition, the use of immortalized mouse lung fibroblast cell lines in certain aspects does not represent an appropriate approach as compared with primary cultures.

The aim of the present study is to develop a curated protocol for the isolation and culture of primary lung fibroblasts from newborn mice using an optimized and highly efficient isolation method. The resulting primary cultures are suitable for 2D studies and provide the required support to epithelial lung cells for 3D lung organoids generation.

## 2. Experimental Design

**Figure 1:**
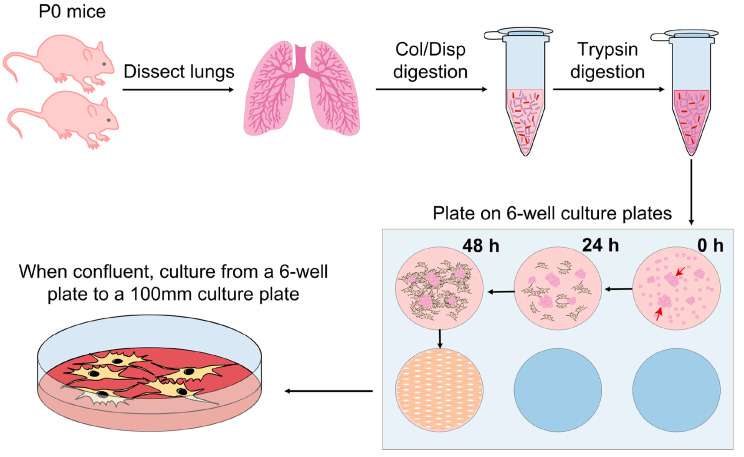
Experimental design of the protocol. Collagenase/Dispase (Col/Disp). Red arrows on 6-well culture plates represent lung tissue pieces from which lung fibroblasts will crawl-out.

The protocol procedures are summarized in Figure 1. Reagents must be prepared using sterile HBSS 1X pH 7.2.

### 2.1. Materials

1. Sterile surgical scissors and forceps;
2. 70% Ethanol;
3. Hank’s Balanced Salt Solution (HBSS) 10X (Gibco, 14175095);
4. Sterile HBSS 1X diluted with MiliQ water and with pH adjusted to 7.2;
5. Sterile phosphate-buffered saline (PBS) 1X;
6. MiliQ water;
7. Collagenase/Dispase^®^ (Roche, 10269638001);
8. 0.25% Trypsin-EDTA (Gibco, 25200-056);
9. Sterile Red Blood Cell lysis (RBC) buffer (0.155 M NH4Cl, 10 mM KHCO3, 10 mM EDTA, pH 7.4);
10. DMEM (Gibco, 41966-029);
11. Foetal Bovine serum (FBS) (Gibco, 10270-106);
12. Penicillin/Streptomycin (P/S) (Gibco, 15140-122);
13. Amphotericin B (Gibco, 15290-026);
14. 6-well cell culture plates;
15. 100 mm cell culture Petri plates;
16. 1.5 mL Eppendorf tubes;
17. Dimethyl sulfoxide (DMSO);
18. Sterile glass coverslips;
19. Fibronectin solution for coating (Sigma, F0556);
20. Bovine serum albumin (BSA) (Sigma, 9048-46-8);
21. Goat serum (Sigma, G9023);
22. Triton X-100 (Sigma, X100);
23. Alexa Fluor™ 488 phalloidin (Invitrogen, A12379);
24. Alpha-smooth muscle actin (GeneTex, GTX100034);
25. Cy3-AffiniPure Goat Anti-Rabbit IgG (H + L) (Jackson ImmunoResearch, 111-165-003);
26. ProLong Diamond antifade reagent (Life Technologies, P36970);
27. NZYol (NZYtech, MB18501);
28. Qiagen RNeasy Mini Kit (Qiagen, 74104);
29. Luna Universal One-Step RT-qPCR kit (New England Biolabs, E3005);
30. 96 or 384-well plate suitable for Real-time PCR System.

### 2.2. Equipment

- Thermo-shaker for Eppendorf tubes;
- Ultracentrifuge for Eppendorf tubes;
- Biological culture hood;
- Cell culture incubator;
- Confocal microscope;
- Nanodrop;
- Real-Time PCR System.

### 2.3. Freshly Prepared Solutions (to Be Made Fresh Immediately before the Experiment)

- Collagenase/Dispase^®^ 1 mg/mL diluted in sterile HBSS 1X. Keep in ice until use.
- Freezing media: 10% DMSO, 20% FBS in DMEM;
- Blocking solution for immunofluorescence staining (5% BSA, 2% goat serum, 1% Triton X-100 in PBS);
- Antibody solution for immunofluorescence staining (2% BSA, 2% goat serum, 1% Triton X-100 in PBS).

## 3. Procedure

### 3.1. Tissue Collection and Dissociation

(Note: all steps should be performed under aseptic conditions in a biological hood. Hereinafter HBSS 1X will be named as HBSS)

**Figure 2:**
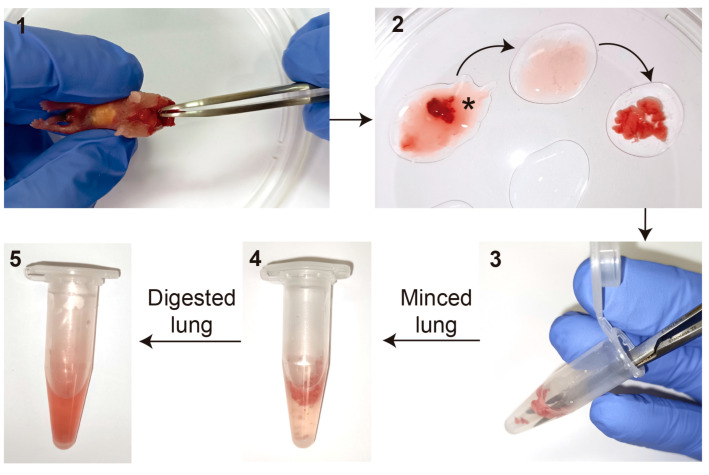
Protocol workflow. (**1**) Lung lobes attached to the heart are dissected from newborn mice without opening the peritoneal cavity. (**2**) Lung lobes in HBSS after elimination of heart, trachea, and other non-lung tissue. Asterisks indicate the discarded no-lung tissue. Arrows indicate the recommended washing steps in drops of HBSS to eliminate blood excess. (**3**) Lung lobes are minced with scissors in a 1.5 mL Eppendorf containing 0.5 mL of the Col/Disp digestion buffer. (**4**) Small pieces of the lungs after mincing. (**5**) Final appearance of fully digested lungs after Col/Disp and Trypsin incubations.

1. Briefly wash newborn mice skin with 70% Ethanol. Euthanize newborn pups by decapitation. Make a small incision with the scissors from the neck to the chest without reaching the peritoneal cavity, and use forceps to remove the lungs attached to the heart; 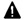 **CRITICAL STEP** For multiple pups, euthanize one after another. Do not reach the peritoneal cavity to avoid contaminations;
2. Place the lung lobes attached to the heart into a drop of HBSS in a sterile 100 mm culture plate containing multiple drops of HBSS. Remove the heart and other non-lung tissue, including the trachea and bronchi, and collect the 5 lung lobes individually one by one. Wash out the blood as much as possible by passing the tissue from drop to drop of HBSS at least 3 times (Figure 2).

### 3.2. Enzymatic Digestion

1. For the first enzymatic digestion, place the lung lobes into a 1.5 mL Eppendorf tube containing 0.5 mL of 1 mg/mL Collagenase/Dispase^®^ and mince thoroughly with small surgical scissors. Incubate at 37 °C for 30 min (min) with gentle agitation;
2. After the incubation, vortex the Eppendorf briefly and centrifuge for 5 min at 1000 × *g* at room temperature (RT). Carefully decant and discard the supernatant; 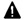 **CRITICAL STEP** The digested lung is viscous and does not remain strongly attached at the bottom of the tube. Thus, to avoid losing material, special care is needed when decanting the supernatant;
3. Wash the pellet by adding 1 mL of HBSS and centrifuge for 5 min at 1000 × *g*. Carefully decant and discard the supernatant;
4. For the second enzymatic digestion, add 0.5 mL of 0.25% Trypsin-EDTA to each Eppendorf and mix thoroughly by pipetting up and down several times. Incubate at 37 °C for 20 min with gentle agitation; 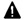 **CRITICAL STEP** Do not exceed 20 min digestion time with trypsin, otherwise, the final cell recovery will be extremely low;
5. Centrifuge for 5 min at 1000 × *g* at RT. Carefully decant and discard the supernatant.

### 3.3. Red Blood Cell (RBC) Removal

1. Resuspend the pellet in 0.1 mL of RBC at RT and incubate for 1 min. Quickly, neutralize by adding 1.2 mL of 1X PBS; 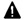 **CRITICAL STEP** If the erythrocytes are not eliminated, the efficiency will be very low. Do not exceed the lysis time, otherwise, the viability of the fibroblasts will be affected;
2. Centrifuge for 5 min at 1000 × *g* at RT. Decant and discard supernatant;

### 3.4. Platting and Freezing

Lung fibroblasts can be maintained as a primary cell culture for up to 6 passages. However, we recommend using a maximum of 4–5 passages.

**Figure 3:**
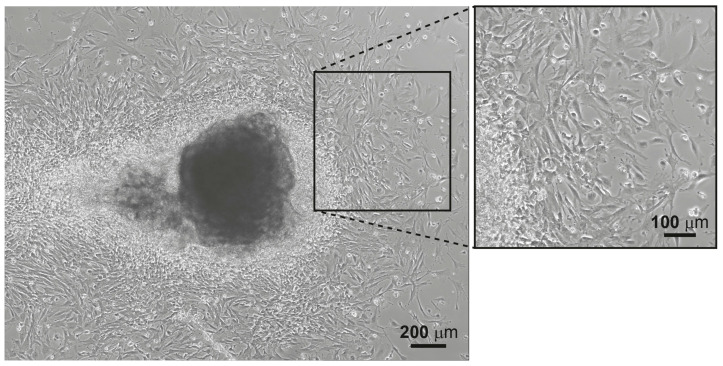
Light microscopy image of lung fibroblasts crawling-out from lung tissue pieces (**left**) and higher magnification image (**right**). Scale bars: 200 and 100 µm for the magnified area.

1. Resuspend the pellet in 0.5 mL of fibroblast culture media (DMEM supplemented with 10% of FBS, 1% P/S, 0.2% Amphotericin B). Gently pipette up and down to break big aggregates and plate the suspension into a 6-well tissue culture plate; 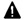 **CRITICAL STEP** Do not discard tissue pieces as lung fibroblasts will crawl out from those pieces, see Figure 3;
2. Add 2 mL of fibroblast culture media so the final volume in each well will be about 2.5 mL;
3. Culture cells at 37 °C and 5% CO_2_;
4. After 24 h, change the media. If erythrocytes are still present in the well, carefully wash with 2 mL of 1X PBS, aspirate PBS and add 2.5 mL of fresh fibroblasts media. After the first change of media, change it every other day. Split to a 100 mm culture plate when the cells are confluent;
5. Freeze 1 vial of cells from 1 confluent 100 mm culture plate (containing about 5 million cells). To detach cells, remove media, wash with sterile PBS to remove FBS residues, aspirate PBS and add 1 mL of 0.25% Trypsin-EDTA. Incubate for 2 min at 37 °C, then, add at least 2 mL of media to the 100 mm culture plate to neutralize the effect of the trypsin;
6. Place the 3 mL of cells into a 15 mL tube and centrifuge for 5 min at 1200 rpm. Remove the supernatant and resuspend the cell pellet in lung fibroblasts freezing media (10% DMSO, 20% FBS in DMEM). Place cryovial swiftly into ice and freeze up to −80 °C using a cell freezing container. After 24 h, place cryovials into liquid nitrogen.

### 3.5. Immunofluorescence (IF)

**Figure 4:**
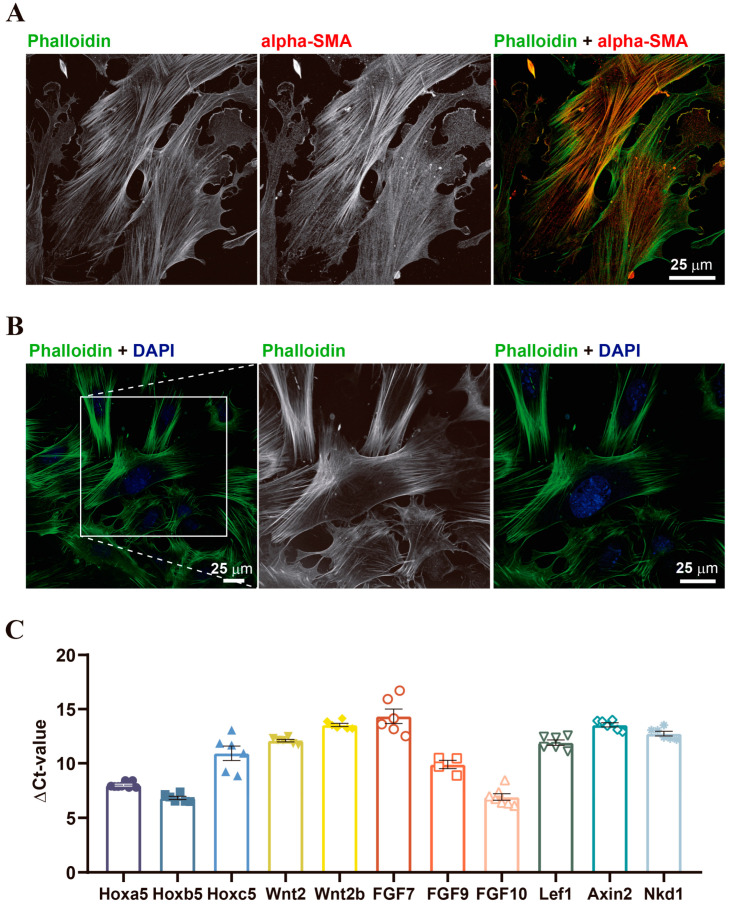
Immunofluorescence staining and RT-qPCRs confirming the purity of the primary lung fibroblast culture. (**A**). Alpha-smooth muscle actin (alpha-SMA) and phalloidin are used as positive markers. Scale bar 25 µm. (**B**). Phalloidin staining with DAPI counterstained nuclei. Scale bars: 25 µm. (**C**). Gene expression in newborn lung fibroblast of specific genes from the Hox-Wnt2-FGF axis.

To characterize the purity of lung fibroblast isolation, IF against alpha-smooth muscle actin or phalloidin staining can be performed, see Figure 4A,B.

1. Place sterile glass coverslips on a 24-well culture plate and coat them with 40 µL of 0.2 µg/mL fibronectin diluted in sterile 1X PBS overnight at 37 °C;
2. Plate 1.5 × 10^4^ cells (cell density must be determined experimentally) on each fibronectin-coated coverslip. Final volume in each well should be approximately 0.5 mL. Incubate 12–24 h in a cell incubator;
3. Aspirate media and wash each well with 1X PBS. Aspirate PBS and fix the cells with 0.5 mL of 4% paraformaldehyde (PFA) for 15 min at 37 °C. Eliminate PFA and wash 2 times with 1X PBS;
4. Permeabilize the cells by washing them for 10 min with 0.1% Triton X-100 in PBS (PBS-Triton);
5. Incubate cells with blocking solution (5% Bovine serum albumin (BSA), 2% goat serum, 1% Triton X-100 in PBS) for at least 30 min at RT;
6. Incubate with the actin primary antibody (1:100) diluted in antibody solution (2% BSA, 2% goat serum, 1% Triton X-100 in PBS) overnight at 4 °C;
7. Wash 3 × 5 min with 1X PBS and permeabilize with PBS-Triton for 10 min. Then, incubate the secondary antibody (Cy3 Goat anti-rabbit, 1:500) together with phalloidin (1:1000) and DAPI diluted in antibody solution for 1 h at RT;
8. Wash 3 × 5 min with 1X PBS, mount coverslips with ProLong Diamond antifading reagent and examine them using a confocal microscope.

### 3.6. qPCR Assays

We further tested the purity of lung fibroblast isolation by qPCRs to detect the expression of specific lung mesenchyme genes (Figure 4C).

1. Grow the cells in a 100 mm culture plate up to 70–80% confluency;
2. Remove the media and wash 2x with 1X PBS. Completely aspirate the PBS and add 0.5 mL of NZYol. Detach and lysate the cells using a cell scraper and collect the sample into a 1.5 mL tube;
3. (Note: at this point, samples can be stored at −80 °C prior to RNA isolation);
4. Isolate RNA with NZYol following the manufacture’s indications, and further purify it using the Qiagen RNeasy Mini Kit columns;
5. RNA concentration and quality can be assessed by RNA capillary electrophoresis columns, or using a Nanodrop;
6. RT-qPCR assays to detect expression levels of genes of interest (Table 1) can be assessed using the Luna Universal One-Step RT-qPCR kit following the manufacture’s protocol. β2-microglobulin is used as a housekeeping gene to have an endogenous control to normalize results.

## 4. Expected Results and Discussion

Established mouse neonatal cell lines for lung fibroblasts are a useful ready-to-use tool in biology, however, to analyze the mesenchyme from a specific neonatal mouse experimental model, the culture of primary lung fibroblasts is recommended. The currently available protocols for adult lung fibroblast isolation are not suitable for the isolation of primary lung fibroblasts from newborn mice, since performing perfusion to eliminate blood cells is not possible in neonate mice. In this regard, eliminating erythrocytes with RBC lysis buffer is a critical step for a successful primary lung fibroblast isolation, although a quick neutralization is required to avoid losing cell viability. Additionally, the combined enzymatic digestion with Collagenase/Dispase^®^ and 0.25% Trypsin-EDTA allows a decrease in the overall digestion time (50 min in total), as well as to perform softer digestion of the lung tissue. This avoids excessive cell death and results in a faster primary culture generation.

In this manuscript, we propose an optimized protocol to establish a rapid primary lung fibroblast culture from newborn mice (approximately fully confluent cultures are achieved within 48–72 h after isolation), as compared to those achievable with previously published protocols for adult fibroblast isolation [16,17]. This study also shows that our protocol generates pure lung fibroblast cultures, as demonstrated through immunofluorescence and gene expression assays. Taken together, these results indicate that our combined enzymatic and crawl-out protocol, together with erythrocyte elimination before plating, leads to a high recovery of lung fibroblasts and will be a useful tool for investigating lung mesenchyme biology.

## Figures and Tables

**Table 1:** Primers used for RT-qPCR assays in lung fibroblasts.

Gene	Accession Number	Sequence
Hoxa5	P09021	F- CAGGGTCTGGTAGCGAGTGT R- CTCAGCCCCAGATCTACCC
Hoxb5	P09079	F- CTGGTAGCGAGTATAGGCGG R- AGGGGCAGACTCCACAGATA
Hoxc5	P32043	F- TTCTCGAGTTCCAGGGTCTG R- ATTTACCCGTGGATGACCAA
Wnt2	P21552	F- TCTTGAAACAAGAATGCAAGTGTCA R- GAGATAGTCGCCTGTTTTCCTGAA
Wnt2b	O70283	F- CTGCTGCTGCTACTCCTGACT R- GGGGATGTTGTCACAGATCA
FGF7	Q544I6	F- CTGCTCCACGCTAACTTCCA R- GAGTTTACGCACCAGCACAC
FGF9	P54130	F- TTCATGCGGTGGGTTCTTATT R- TCCTCATCCAAGCTTCCATCA
FGF10	O35565	F- GTCAGCGGGACCAAGAATGA R- GTCGTTGTTAAACTCTTTTGAGCC
Lef1	P27782	F- AAATGGGTCCCTTTCTCCAC R- CTCGTCGCTGTAGGTGATGA
Axin2	O88566	F- CAGTGAGCTGGTTGTCACCT R- TCCTCAAAAACTGCTCCGCA
Nkd1	Q99MH6	F- TAGACCTGGCGGGGATAGAG R- GTCAAGGAGGTGGAAGGAGC

## Data Availability

No new data were created or analyzed in this study. Data sharing is not applicable to this article.
